# Functional Group Effects on the Photoelectronic Properties of MXene (Sc_2_CT_2_, T = O, F, OH) and Their Possible Photocatalytic Activities

**DOI:** 10.1038/s41598-017-15233-8

**Published:** 2017-11-08

**Authors:** Kuangwei Xiong, Peihong Wang, Guang Yang, Zhongfei Liu, Haijun Zhang, Shaowei Jin, Xin Xu

**Affiliations:** 1 School of Physics and Materials Science, Anhui Key Laboratory of Information Materials and Devices, Anhui University, Hefei, 230601 PR China; 2 Department of Physics, East China Jiaotong University, Nanchang, 330013 PR China; 3 College of Energy and Environmental Engineering, Hebei University of Engineering, Handan, 056038 Hebei Province PR China; 4 School of Mathematics Science, Anhui University, Hefei, 230601 PR China

## Abstract

In view of the diverse functional groups left on the MXene during the etching process, we computationally investigated the effects of surface-group types on the structural, electronic and optical properties of Sc_2_CT_2_ (T = -O, -OH, -F) MXenes. For all geometries of the Sc_2_CT_2_ MXenes, the geometry I of Sc_2_CT_2_, which has the functional groups locating above the opposite-side Sc atoms, are lowest-energy structure. Accordingly, the energetically favorable Sc_2_CF_2_-I, Sc_2_CO_2_-I and Sc_2_C(OH)_2_-I were selected for further evaluation of the photocatalytic activities. We found that the Sc_2_CO_2_-I is metallic, while Sc_2_CF_2_-I and Sc_2_C(OH)_2_ are semiconductors with visible-light absorptions and promising carrier mobilities. Compared with the Sc_2_C(OH)_2_-I, the Sc_2_CF_2_-I has not only more suitable band gap (1.91 eV), but also the higher redox capability of photo-activated carriers, which should have better photocatalytic performance.

## Introduction

Since the discovery of graphene by Novoselov *et al*.^1^, two-dimensional (2D) materials have garnered tremendous interest in experimental and theoretical studies due to the unique properties of these 2D free-standing crystals. Several families of 2D materials have been recently identified and investigated, such as transition metal dichalcogenides (TMDs)^2^, hexagonal boron nitrides^3^, few-layer metal oxides^4^, metal chalcogenides (e.g. MoS_2_^5^,WS_2_^6^). Remarkably, the constellation of 2D materials has been augmented by a potentially quite large group of early transition metal carbides and carbonitrides (MXenes)^7^. MXenes was generally fabricated by selectively etching “A” layers from M_n+1_AX_n_ phases through acid solution etching^8–12^. Here, “M” is an early transition metal, “A” is mainly a group IIIA or IVA element (e.g. Al, Si), X is C and/or N, n = 1, 2, or 3.

During the etching process, “A” atoms were removed and functional groups (-O, -OH, or -F) were left on the surface of MXene to passivate the outer-layer metal atoms. MXene phases present a number of configurations which have variety of novel properties according to the species of functional groups. Many theoretical studies show that bare MXene are metallic and the majority of MXene are passivated by -OH, -F and -O groups still remain metallic. Exceptionally, some of the M_2_C (M = Ti, Zr, Hf, Sc,) MXenes turn from metallic to semiconducting by surface functionalization (-O, -OH, or -F), which have band gaps between 0.24 ∼ 1.8 eV^13^. The suitable band gaps of surface functionalized MXenes strongly indicate their visible-light absorption and possible photocatalytic applications.

Compared with other 2D materials, MXenes possess many exceptional properties for photocatalytic application. For example, the promising electric conductivity and carrier mobility could promote the migration of photo-induced carriers, resulting in more effective participation of e^-^-h^+^ pairs during the photocatalytic process. The surface hydroxy group may serve as the anchor site for the reactants, resulting in more efficient adsorption and oxidation of the organic molecules. The band gaps of the functionalized MXenes are expected to be 0 ~ 2 eV, which reach the requirements of efficient visible-light absorption. Additionally, experimental studies of MXenes have mainly focused on their practical application of lithium-ion batteries, supercapacitors, and adsorbents, few results have been achieved on their photocatalytic utilization. In view of the above reasons, it is quite indispensable to evaluate the possibilities of photocatalytic application, in order to further explore the potential utilizations of these novel graphene-like materials.

In our previous work^14^, we theoretically investigated the electronic and optical properties of functionalized MXenes M_2_CT_2_ (M = Ti, Zr, Hf, T = -O, -OH, -F) with different functionalization geometries. Among the variously functionalized MXenes, only M_2_CO_2_ (M = Ti, Zr, Hf) with geometry I, which have the functional group located above the opposite-side metal atoms, have appropriate band gaps (0.92 ∼ 1.75 eV) for photocatalytic applications. Regarding to the Sc_2_CT_2_ (T = -O, -OH, -F) MXenes, first-principle calculations^13^ by using the Generalized Gradient Approximation (GGA) in the form of Perdew-Burke-Ernzerhof (PBE) functional found that the Sc_2_C(OH)_2_ has a direct band gap of 0.45 eV, while the Sc_2_CF_2_ and Sc_2_CO_2_ have indirect band gaps of 1.03 and 1.80 eV, respectively. As we known, the band gaps of these MXenes are underestimated due to the PBE functional. The hybrid functional of Heyd-Scuseria-Ernzerhof (HSE06) method was proven a reliable method in the calculation of electronic structures^15,16^. Moreover, the effects of surface group on the electronic and optical properties of Sc_2_CT_2_ (T = -O, -OH, -F) MXenes and the possibilities of their photoelectronic applications are yet to be addressed.

In this paper, the first-principle calculations were performed to explore the structural, electronic and optical properties of Sc_2_CT_2_ (T = -O, -OH, -F) and to further discuss their probability for photocatalytic applications. The crucial characteristics of these Sc_2_CT_2_ were summarized, including the thermodynamic stabilities, band structures, optical absorptions, redox potentials and carrier mobility. The computational results suggest that Sc_2_CF_2_-I with -F group locating at the opposite position of outer metal atoms have appropriate band gaps of 1.91 eV, visible-light absorption and strong redox capability of photo-induced excitons. Based on our calculation results, the fluorinated Sc_2_C MXene could be a promising candidate for high-performance photocatalysts.

## Results

### Thermodynamic stabilities of Sc_2_CT_2_ (T = -O, -OH, -F) MXenes

We firstly check the relative stabilities of the optimized MXenes with different geometries. The total energies (*E*_*tot*_) of the optimized MXenes are listed in Table 1. In general, our results are consistent with previous report^13^. In three geometries, geometry-I structures of Sc_2_CO_2_, Sc_2_C(OH)_2_, Sc_2_CF_2_ usually have the lowest total energy, which demonstrates that geometry I is energetically more favorable than geometry II and III.

**Table 1:** Total energy (*E*_*tot*_ in eV/unit cell) and Cohesive energies (*E*_*coh*_ in eV/atom) of the functionalized Sc_2_CT_2_ (T = -O, -OH, -F) MXenes.

MXenes	Total energy *E*_*tot*_			Cohesive energies *E*_*coh*_		
geometry	Sc_2_CO_2_	Sc_2_C(OH)_2_	Sc_2_CF_2_	Sc_2_CO_2_	Sc_2_C(OH)_2_	Sc_2_CF_2_
I	**−40**.**58**	**−49**.**13**	**−37**.**88**	**6**.**38**	**5**.**46**	**6**.**25**
II	−39.24	−48.36	−36.67	6.11	5.35	6.01
III	−39.90	−48.77	−37.33	6.24	5.41	6.14

The most stable geometry is highlighted in bold.

We further calculated the cohesive energy to evaluate the stabilities of the investigated MXenes. The cohesive energy (*E*_*coh*_) were defined as:

1 $${E}_{coh}=(2n{E}_{Sc}+n{E}_{C}+2n{E}_{T}\,-\,n{E}_{S{c}_{2}C{T}_{2}})/N$$

where *E*_*Sc*_, *E*_*C*_, are the total energies of a single Sc atom and C atom, *E*_*T*_ is that of a single O, F atom or one O atom plus H atom, ${E}_{S{c}_{2}C{T}_{2}}$ denotes the total energy of one unit cell of Sc_2_CT_2_ monolayer. *N* is the number of atoms in the supercell. The results of cohesive energy calculations are presented in Table 1. According to our results, the cohesive energies of the investigated Sc_2_CT_2_ MXene range from 5.35 to 6.38 eV/atom, which are comparable to those of FeB_6_ monolayer (5.56 ∼ 5.79 eV/atom)^17^, Al_2_C monolayer has (4.49 eV/atom)^18^, and Be_2_C monolayer (4.86 eV/atom)^19^. This indicates that the formation of strong chemical bonds between the Sc atom and T (T = -O, -OH, -F) groups. In view of the comparable *E*_coh_ values of Sc_2_C*T*_2_ MXenes (5.35 to 6.38 eV) and FeB_6_ monolayers (5.56 ~ 5.79 eV), it is proposed that the Sc_2_CT_2_ have the same type of chemical bonds with the FeB_6_ monolayer, in which there is obvious electron transition from the Fe atom to B framework, and thus the ionic bonding between the Fe and B atoms. Therefore, the high *E*_coh_ values of Sc_2_CT_2_ MXenes also suggest the strong ionic bonds within them.

Generally, the structure with lower total energy usually imply the higher cohesive energy of the corresponding structure. In other word, the highest-cohesive-energy geometry should have the lowest total energy, and should be the energetically preferable structure. As shown in Table 1, The *E*_*coh*_ values increase with functional group in the order of Sc_2_CO_2_ > Sc_2_CF_2_ > Sc_2_C(OH)_2_. The largest *E*_*coh*_ values for O functionalized Sc_2_C may due to the stronger interaction between the O and Sc atom, which results from the shorter bond length of Sc-O (2.00 Å) than those of Sc-F (2.21 Å) and Sc-OH (2.27 Å). The same trend holds for most of other transition metal carbides (e.g Ti, V, Cr, Mo, Hf)^20^ Moreover, *E*_*coh*_ values are also affected by the geometric structure. The cohesive energies of Sc_2_CT_2_ MXenes are in the decreasing order of Geometry-I > Geometry-III > Geometry-II. The highest cohesive energy of Geometry-I indicates that Geometry-I is the most beneficial to the formation of strong chemical bonds in the Sc_2_CT_2_. With a definite functional group, the preference of Geometry-I rather than Geometry-II and Geometry-III is affected by several factors such as the number of electrons demanded by the functional groups, the hybridization strength between the functional groups and transition metal^13^.

### Electronic structures and optical properties

**Figure 1:**
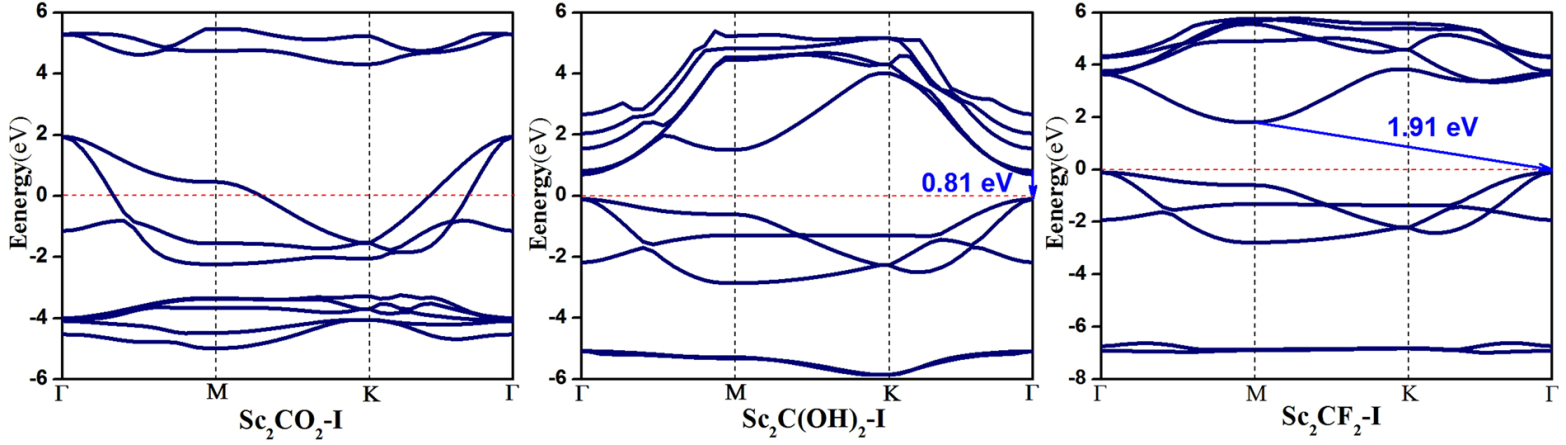
Band structures of the functionalized MXenes with geometry I. The Fermi level is at 0 eV (the red dashed lines).

According to the above results of cohesive energies, Geometry-I of Sc_2_CO_2_, Sc_2_C(OH)_2_, Sc_2_CF_2_ is the most stable structure. Therefore, we only calculated the electronic structures and optical properties of Sc_2_CO_2_-I, Sc_2_C(OH)_2_-I and Sc_2_CF_2_-I in order to find out whether they have appropriate characteristics for photocatalytic applications. Based on the HSE06 hybrid functional, the band structures of three Sc_2_CT_2_ (T = -O, -OH, -F) MXenes with geometry I are computed and presented in Fig. 1. We found that Sc_2_CO_2_-I is metallic, while the Sc_2_C(OH)_2_-I and Sc_2_CF_2_-I materials are semiconductors. Accordingly, the latter two MXene were selected for further investigation. The Sc_2_C(OH)_2_ presents a direct band gap of 0.81 eV with the valence band maximum (VBM) and the conduction band minimum (CBM) both located at Γ point, while Sc_2_CF_2_-I has an indirect band gap of 1.91 eV with the VBM located at Γ point and CBM at M point, which agree well with the previous computational results^20,21^. The indirect band gap is beneficial to the restraint of electron-hole recombination in the photocatalytic reaction^22,23^.

HSE06 hybrid functional combines one-quarter of the exact Hartree–Fock exchange energy with three-quarters of an approximate exchange-correlation energy. This combination in general produces more reliable band gaps than GGA-PBE. As expected, the values of band gaps obtained by our HSE06 computations are greater than those of Sc_2_C(OH)_2_ (0.45 eV) and Sc_2_CF_2_ (1.03 eV) in other calculation based on PBE functionals^13^. For Sc_2_CO_2_ materials, the Geometry-I structure with the lowest energy was selected for the comparison in our work, while the band structure of Geometry-III with the second lowest energy was computed in ref.^13^. Accordingly, the metallic property of Sc_2_CO_2_ in our work is not in accordance with the results in ref.^13^, in which the Sc_2_CO_2_ is semiconductor. The lowest energy of the Geometry-I indicates its best thermodynamic stability and the highest experimental feasibility, and accordingly, should be selected for the further evaluation of the photocatalytic activities.

**Figure 2:**
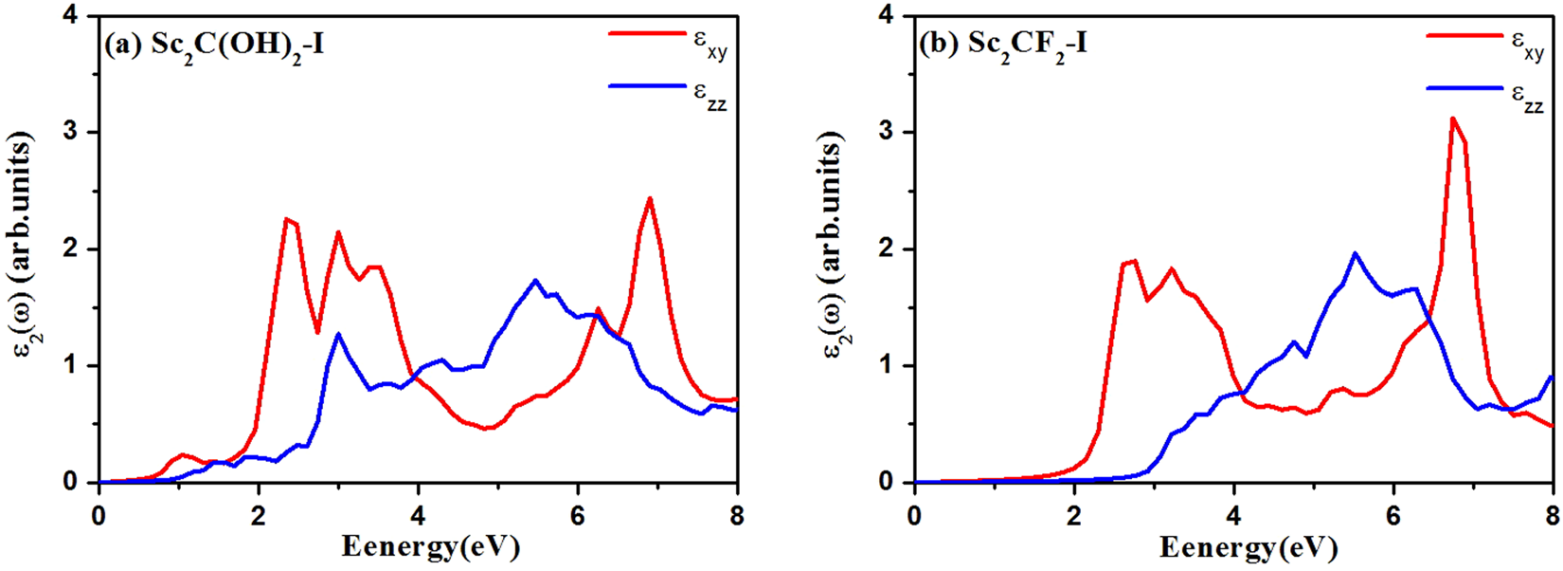
Imaginary parts of dielectric constants of the Sc_2_C(OH)_2_-I and Sc_2_CF_2_-I.

We also calculated the dielectric constants $\varepsilon (\omega )={\varepsilon }_{1}(\omega )+i{\varepsilon }_{2}(\omega )$ of Sc_2_C(OH)_2_-I and Sc_2_CF_2_-I to further confirm their optical absorption properties. The imaginary parts ${\varepsilon }_{2}(\omega )$ of the dielectric constants consists of ${\varepsilon }_{xy}(\omega )$ and ${\varepsilon }_{zz}(\omega )$, which are the components perpendicular and parallel to the z direction, respectively. We can see from Fig. 2 that the Sc_2_C(OH)_2_-I has three obvious absorption peaks at 1.04 eV, 2.34 eV and 2.99 eV in the visible-light region (<3.0 eV). The imaginary part of Sc_2_CF_2_-I has one peak at 2.76 eV. The Sc_2_CF_2_-I also has obvious absorption in the visible-light region (1.99 eV to 2.76 eV), implying the harvest of the major portion of visible light. These evidences of the absorption spectra indicate their potential application in visible light driven photocatalysis and other photoelectronic process.

**Figure 3:**
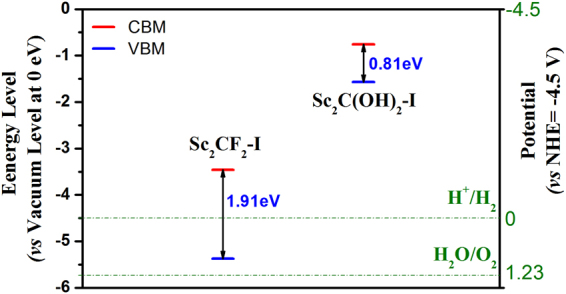
Band edge position of the Sc_2_C(OH)_2_-I and Sc_2_CF_2_-I. The redox potentials of H^+^/H_2_ and H_2_O/O_2_ at PH = 0 are also provided as a reference (the green dashed lines).

From the viewpoint of photoelectrochemistry, the photocatalytic activity of the materials is also affected by the band edge position. The band edge positions were obtained by calculating the CBM and VBM energies relative to the vacuum level at 0 eV in our computations. Additionally, in order to evaluate the oxidation/reduction ability of the investigated materials, a normal hydrogen electrode (NHE) is selected as a reference, which potential is equal to −4.5 V with respect to the vacuum level (Fig. 3 and Table S1). Generally, the photocatalytic material should have photo-induced electrons with the stronger reduction capabilities if the CBM level is more negative and photo-activated holes in it should have the greater oxidation capabilities if the VBM energy is more positive.

As represented in Fig. 3, Sc_2_C(OH)_2_-I has a narrow band gap of 0.81 eV, which has absorption peak at the energies of 1.04 eV, 2.34 eV and 2.99 eV. However, the tiny band gap of it may result in the easier transition of the activated electrons from CBM to VBM, which lead to the fast recombination of photo-induced e^-^ -h^+^ pairs^16^. The Sc_2_C(OH)_2_-I with narrow bandgap may be utilized to form composites with other wide gap semiconductors, just like the graphene/TiO_2_^24^ and graphene/ZnO nanocomposites^25^. These composites with well-matched band structures can broaden light absorption and enhance the separation of photo-generated electrons and holes. Compared to other composite structure^26–28^, in which the great achievements have been obtained, the Sc_2_C(OH)_2_-I MXene may have its unique superiority. For instance, the -OH group at the surface of Sc_2_C(OH)_2_ MXene may be promising active sites for the effective adsorption of reactants, such as the organic molecules and heavy metal ions. The high carrier mobility and good electric conductivity of Sc_2_C(OH)_2_-I could lead to the efficient migration of the photo-activated electron and holes, which would effectively participate in the photoreaction on the surface of composite-structure photocatalysts.

The CBM position of the Sc_2_CF_2_-I is around −1.038 V (*vs*. NHE at PH = 0), which is more negative than the reduction potential of carbon dioxide (CO_2_) into hydrocarbons, such as methane (CH_4_), methanol (CH_3_OH), formic acid (HCOOH). It is also more negative than the potential of H_2_ evolution (0 V *vs*. NHE at PH = 0)^16^. Therefore, the Sc_2_CF_2_-I can be used for photocatalytic reduction of CO_2_ or photocatalytic generation of hydrogen.

In semiconductors, the valence-band holes that have chemical potential of +1.0 to +3.5 V (*vs*. NHE) are powerful oxidants, while the conduction-band electrons with chemical potential of +0.5 to −1.5 V (*vs*. NHE) are good reductants^29^. The VBM (*vs*. NHE) of the Sc_2_CF_2_-I MXene is 0.87 V. So the Sc_2_CF_2_-I MXene also has oxidation capacity at a certain extent. Furthermore, the band edge position can be effectively modified by approaches of Doping^30,31^, tensile strain^32^ and heterojunctions^33^ to reach the requirement of the photocatalytic process.

### Migration mechanism of the carrier

**Figure 4:**
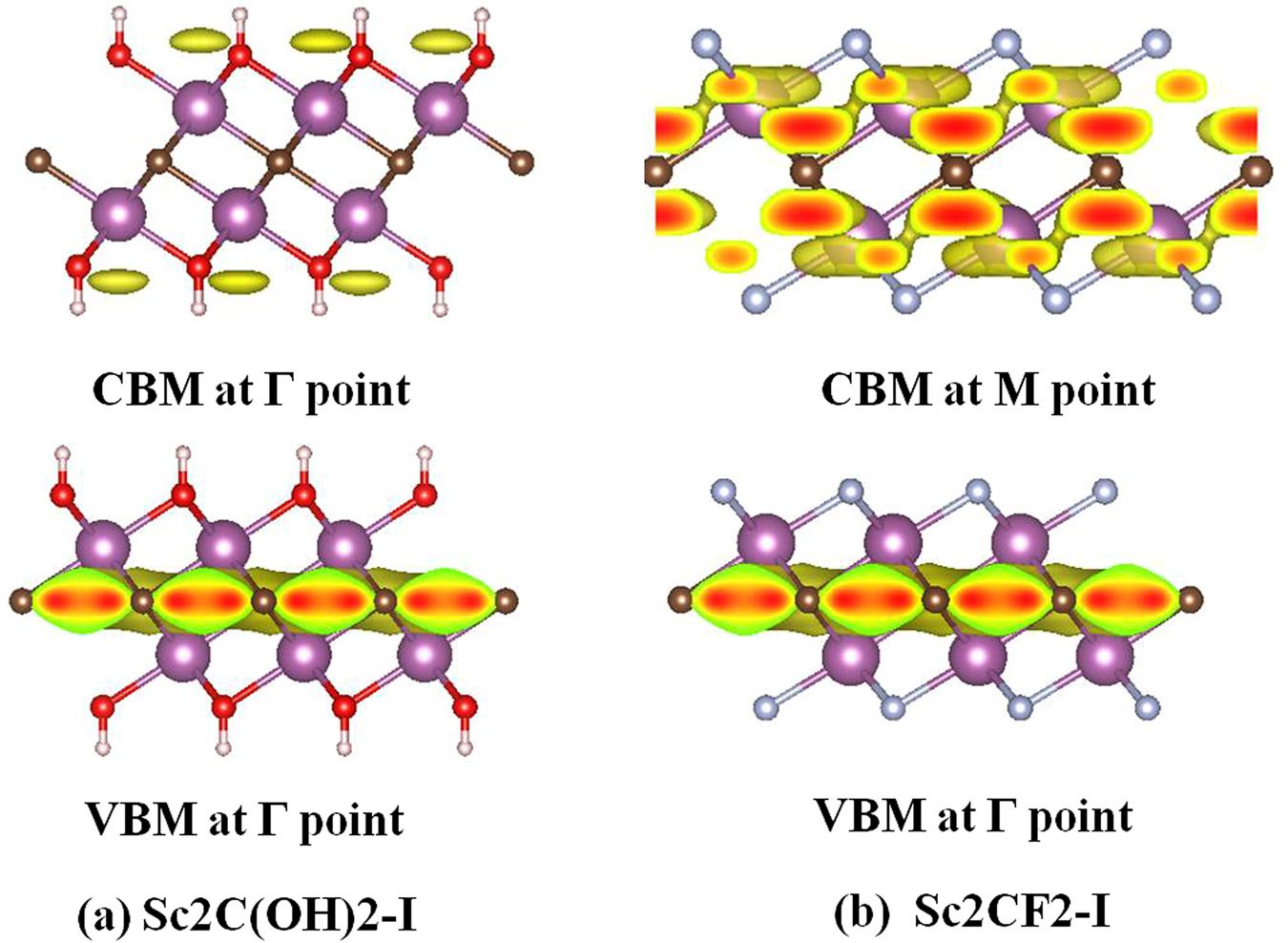
Spatial charge distributions for (**a**) Sc_2_C(OH)_2_-I (**b**) Sc_2_CF_2_-I.

It is necessary to calculate the spatial charge distribution because most photo-induced electrons and holes will transfer to spatial locations of CBM and VBM, respectively. Figure 4a shows the spatial charge distribution of the Sc_2_C(OH)_2_-I. the CBM predominantly distribute around OH layer, while the VBM are located at the carbon layer. Regarding to the spatial charge distribution of the Sc_2_CF_2_-I in Fig. 4b, CBM mostly distribute around Sc atoms, the distribution of VBM is similar to that of the Sc_2_C(OH)_2_-I. These different locations of CBM and VBM could lead to spatial separation of photo-activated electron and holes and inhibit the recombination of electron-hole pairs.

We also computed the electron localization function (ELF)^34^ of the Sc_2_C(OH)_2_-I and Sc_2_CF_2_-I to analyze their electron distributions. Since the electron localization function (ELF)^34^ of the Sc_2_C(OH)_2_-I and Sc_2_CF_2_-I is similar, we only list the result of the Sc_2_CF_2_-I for analysis. From ELF map in Fig. S2, the red region around the C atoms indicates that the electrons are mainly located around the C atoms and there is remarkable electron transfer from Sc to C atoms. As a consequence, C atoms can act as the negative charge center and efficiently capture the photo-activated holes in Sc_2_CF_2_-I material, which results in the efficient separation of photo-generated e^-^ -h^+^ pairs.

**Figure 5:**
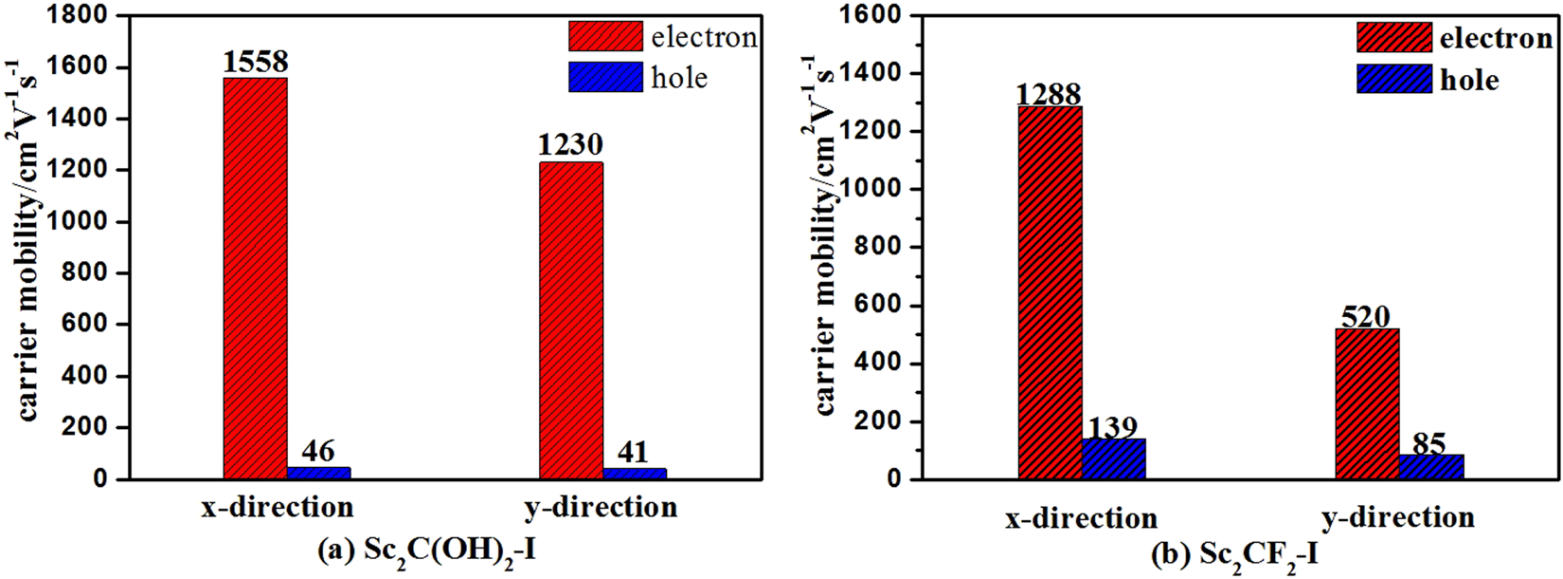
The carrier mobility of the Sc_2_C(OH)_2_-I and Sc_2_CF_2_-I along x-direction and y-direction.

The carrier mobility is another important factor affecting photocatalytic activity. We calculated the carrier mobility of the Sc_2_C(OH)_2_-I and Sc_2_CF_2_-I along x-direction and y-direction on the basis of the hexagonal cell (Fig. S3). The results of the carrier mobility are shown in Fig. 5 and the related calculation details are presented in Fig. S4 and Table S2. As shown in Fig. 5, we found that the electron mobilities of Sc_2_C(OH)_2_-I and Sc_2_CF_2_-I are 1558 cm^2^ V^−1^ s^−1^ and 1288 cm^2^ V^−1^ s^−1^ along x direction, as well as 1230 cm^2^ V^−1^ s^−1^, 520 cm^2^ V^−1^ s^−1^ along y direction, respectively, which are comparable to those of monolayer phosphorene (1140 cm^2^ V^−1^ s^−1^ along x direction and 80 cm^2^ V^−1^ s^−1^ along y direction)^27^, Ti_2_CO_2_ monolayer MXene (611 cm^2^ V^−1^ s^−1^)^35^, and MoS_2_ (about 200 cm^2^ V^−1^ s^−1^)^36^. These results reveal the photo-induced electron can quickly migrate to the active site of photocatalytic reaction on the investigated MXenes.

Furthermore, in both the Sc_2_C(OH)_2_-I and Sc_2_CF_2_-I, the electron mobility is much higher than the hole mobility in x or y direction, which can be ascribed to the faster migration of electrons within the Sc_2_C(OH)_2_-I and Sc_2_CF_2_-I. The great difference between the mobility of electron and holes should result in different transport efficiency of photo-activated electron and holes within these materials. For this reason, when the photo-induced electrons reach the surface, the holes should still stay in the inner layer. This property should be valuable for the separation of electrons and holes during the photocatalytic process.

## Discussion

By using first-principle computation, we have systematically investigated the functional-group-dependent structural, electronic and optical properties of Sc_2_CT_2_ (T = -O, -OH, -F) MXenes. We found that geometry-I of Sc_2_CT_2_ is the most stable one in all three geometries, which is selected to the further studies on the evaluation of their photocatalytic activity. The Sc_2_CO_2_-I is metallic, while the Sc_2_C(OH)_2_-I and Sc_2_CF_2_-I are semiconductors with band gaps of 0.81 eV and 1.91 eV, respectively. Both the Sc_2_C(OH)_2_-I and Sc_2_CF_2_-I exhibit very good optical absorption in the visible-light region. The presence of absorption peaks in the visible light region indicates their potential application as photocatalysts.

Additionally, the Sc_2_CF_2_-I MXene has an excellent reduction potential of −1.038 V (*vs*. NHE) and could be used for photocatalytic reduction of CO_2_ or for photocatalytic hydrogen generation. The photo-induced holes within it is also a good oxidant with redox potential of 0.87 V (*vs*. NHE). Through further studying the migration mechanism of the carrier, we confirmed that both Sc_2_C(OH)_2_-I and Sc_2_CF_2_-I have the efficiently separated electron-hole pairs and good carrier mobility. Relatively, the Sc_2_CF_2_-I can be more conducive to the visible-light photocatalysis due to its more suitable band gap and band edge alignment. Our results provide some fundamental data on the possible MXene photocatalysts, which is helpful in further exploring the practical applications of MXenes.

### Computational Method

For the first-principles calculations, the Vienna Ab-initio Simulation Package (VASP) were employed^37^. The GGA with the PBE^38^ functional was adopted for the exchange-correlation functional. The ion-electron interaction was treated by the projector augmented wave (PAW) method^39^. The energy cutoff was set to 600 eV and the energy precision of the computations was 10^−5^ eV. The atomic positions were fully relaxed until the maximum force on each atom was less than 10^−3^ eV/Å. In the geometry optimization and self-consistent computations, a 9 × 9 × 1 Monkhorst-Pack k-point grid centered on the Ã point was used for the Brillouin zone. All of the band structures were calculated with the HSE06 hybrid functional^40^. The electronic structures were visualized using the VESTA code^41^.

The optical absorption properties of the Sc_2_CT_2_ were investigated by computing the complex dielectric constants (ε) at a given frequency (ω) by using the HSE06 hybrid functional with a 21 × 21 × 1 k-point mesh. The dielectric constants can be defined as $\varepsilon (\omega )={\varepsilon }_{1}(\omega )+i{\varepsilon }_{2}(\omega )$. The absorption coefficient *I*(*ω*) can be calculated through following equation^42^:

2 $$I(\omega )=\sqrt{2}\omega {[\sqrt{{\varepsilon }_{1}{(\omega )}^{2}+{\varepsilon }_{2}{(\omega )}^{2}}+{\varepsilon }_{1}(\omega )]}^{1/2}$$

As shown in the equation (1), only if the imaginary part ${\varepsilon }_{2}(\omega ) > 0$, $I(\omega )$ will be above zero. Therefore, the imaginary part ${\varepsilon }_{2}(\omega )$ reflects the light absorption at a given frequency. The imaginary part can be determined as^43^:

3 $$\begin{array}{rcl}{\varepsilon }_{\alpha \beta }^{(2)}(\omega ) & = & \frac{4{\pi }^{2}{e}^{2}}{{\rm{\Omega }}}\mathop{{\rm{lim}}}\limits_{q\to 0}\frac{1}{{q}^{2}}\sum _{c,\nu ,\mathop{k}\limits^{\rightharpoonup }}2{\omega }_{\mathop{k}\limits^{\rightharpoonup }}\delta ({\varepsilon }_{c\mathop{k}\limits^{\rightharpoonup }}-{\varepsilon }_{\nu \mathop{k}\limits^{\rightharpoonup }}-\omega )\\  &  & \times \langle {u}_{c\mathop{k}\limits^{\rightharpoonup }+{e}_{\alpha }\mathop{q}\limits^{\rightharpoonup }}| {u}_{\nu \mathop{k}\limits^{\rightharpoonup }}\rangle {\langle {u}_{c\mathop{k}\limits^{\rightharpoonup }+{e}_{\beta }\mathop{q}\limits^{\rightharpoonup }}| {u}_{\nu \mathop{k}\limits^{\rightharpoonup }}\rangle }^{* }\end{array}$$

here the indices *c* and *í* are restricted to the conduction and valence band states and ${u}_{c\mathop{k}\limits^{\rightharpoonup }}$ is the cell periodic part of the wavefunctions at the k-point.

The carrier mobility of the Sc_2_CT_2_ was calculated which is based on deformation potential theory^44^ with the following Equation^45–47^:

4 $${\mu }_{2D}=\frac{2e{\hslash }^{3}C}{3{k}_{B}T{| {m}^{* }| }^{2}{E}_{1}^{2}}$$

here the temperature of T = 300 K was adopted in this study; e, $\hslash$ and *k*_*B*_ are electron charge, the reduced Planck constant and the Boltzmann constant, respectively. C is the elastic modulus under the uniaxial strain along the transport direction, given by $C=({\partial }^{2}{E}_{total}/\partial {\varepsilon }^{2})/{S}_{0}$, where ${E}_{total}$ is the total energy of a unit cell under different uniaxial strain å and *S*_0_ is the area of the unit cell in the *xy* plane; *m* ^*^ is the effective mass of the carrier along the transport direction, which is determined by ${m}^{* }={\hslash }^{2}/({\partial }^{2}E(k)/\partial {k}^{2})$; *E*_1_ is the deformation potential (DP) constant of VBM for holes and CBM for electrons, calculated by using ${E}_{1}=\partial {E}_{edge}/\partial \varepsilon$. *E*_*edge*_ is the energy of VBM or CBM along the transport direction.

**Figure 6:**
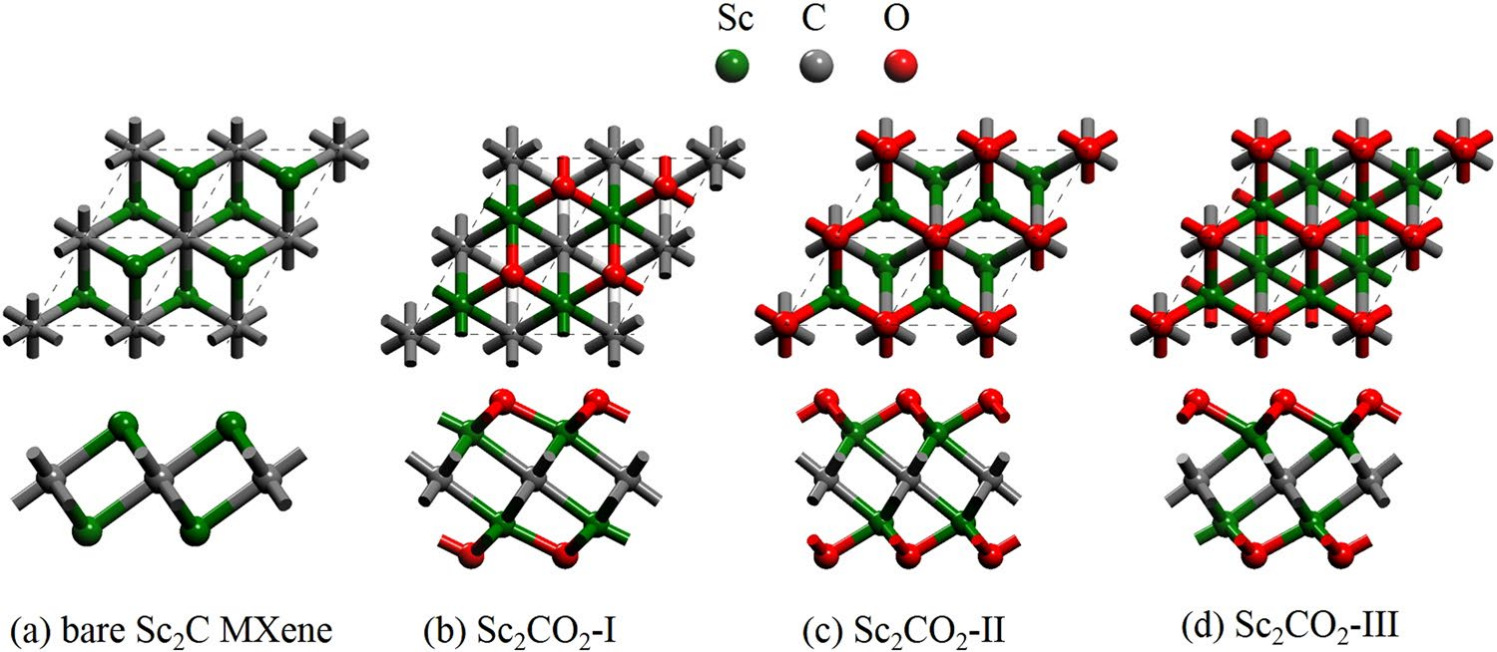
Top view (upper) and side view (lower) of the geometries for (**a**) bare Sc_2_C; (**b**) Sc_2_CO_2_-I; (**c**) Sc_2_CO_2_-II; (**d**) Sc_2_CO_2_-III.

It was well identified in previous works^13,48^ that each functionalized Sc_2_C MXene has three different geometries on its surface. We take the Sc_2_CO_2_ as an example to illustrate them. The top view and side view of Sc_2_CO_2_ structure are shown in Fig. 6, Sc_2_C(OH)_2_ and Sc_2_CF_2_ are shown in Fig. S1 (Supporting Information). As seen in Fig. 1a, Bare Sc_2_C MXene has one carbon layer sandwiched by two scandium layers. The functionalized Sc_2_C MXenes have three configurations: the geometry I with surface O atoms located above the opposite-side Sc atoms (Fig. 6b, Sc_2_CO_2_-I), the geometry II with two terminated O atoms located on the middle C atoms (Fig. 6c, Sc_2_CO_2_-II), as well as the geometry III with one terminated O atom lies on the top of the opposite Sc atom and another one lies on the top of the middle C atom (Fig. 6d, Sc_2_CO_2_-III). A vacuum space of 20 Å was inserted to avoid any interaction between MXene layers along the *z* axis.

## Electronic supplementary material

Supporting Information
