# Supplementary material for: Functional Group Effects on the Photoelectronic Properties of MXene (Sc2CT2, T = O, F, OH) and Their Possible Photocatalytic Activities
Source: Sci Rep. 2017 Nov 8;7:15095. doi: 10.1038/s41598-017-15233-8 (PMC5678094; doi:10.1038/s41598-017-15233-8)
Supplement: Supplementary file 1 — Supporting Information [file 41598_2017_15233_MOESM1_ESM.pdf]

Supporting Information

Functional Group Effects on the Photoelectronic Properties of MXene (Sc_2CT_2, T = O, F or OH) and Their Possible Photocatalytic Activities

Kuangwei Xiong[1,2], PeihongWang[1], Guang Yang[3], Zhongfei Liu[1], Haijun Zhang[1,*], Shaowei Jin[1,*], Xin Xu[4]

[1]School of Physics and Materials Science, Anhui Key Laboratory of Information Materials and Devices, Anhui University, Hefei 230601, PR China

[2]Department of Physics, East China Jiaotong University, Nanchang 330013, PR China

[3]College of Energy and Environmental Engineering, Hebei University of Engineering, Handan 056038, Hebei Province, PR China

[4]School of Mathematics Science, Anhui University, Hefei 230601, China

E-mail: hjzhang_ahu@163.com; jinsw@mail.ustc.edu.cn.

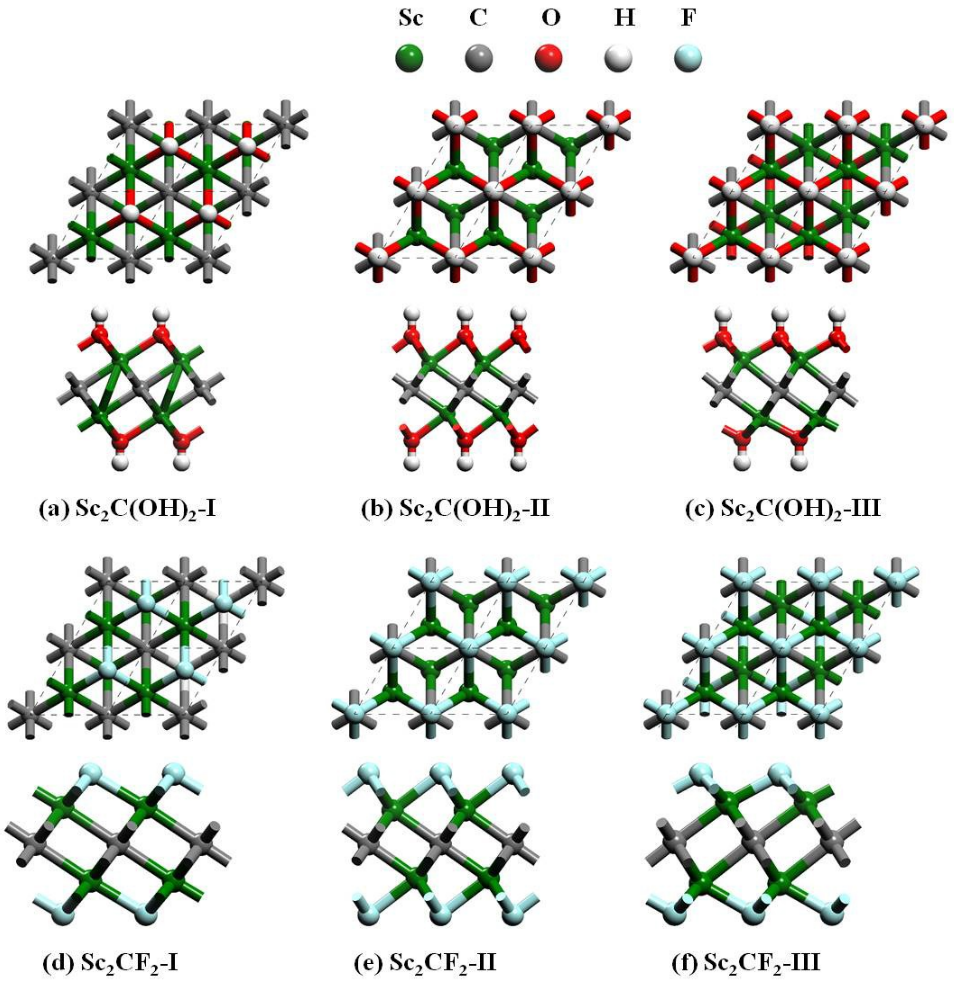

Fig S1 Top view (upper) and side view (lower) of the geometries for (a-c) $Sc_2C(OH)_2$-I, II, III; (d-f) Sc_2CF_2-I, II, III.

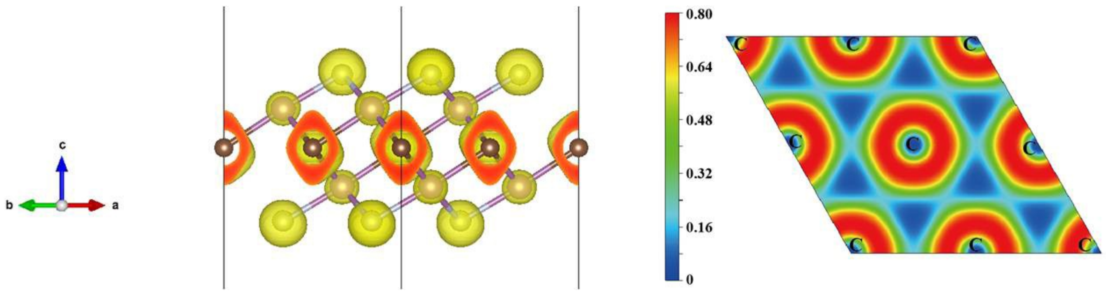

Fig S2 ELF isosurfaces (left) plotted with the value of 0.75 au and ELF map (right) for the Sc_2CF_2-I. In the ELF map, the red and blue represent the highest (0.8) and lowest value (0) of ELF.

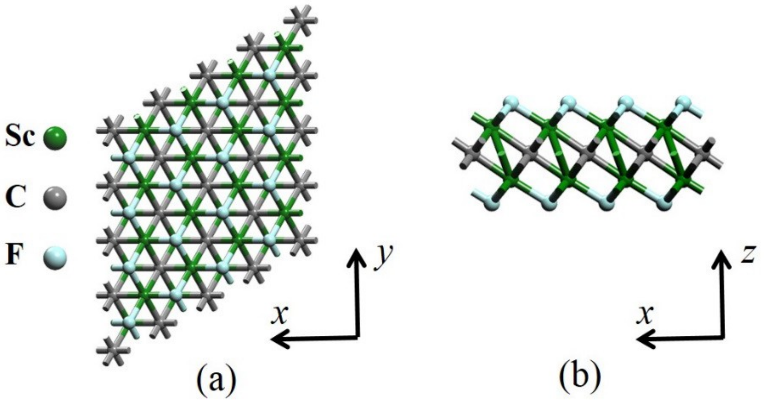

Fig S3 a 2×2 supercell of the Sc_2CF_2-I monolayer MXene.

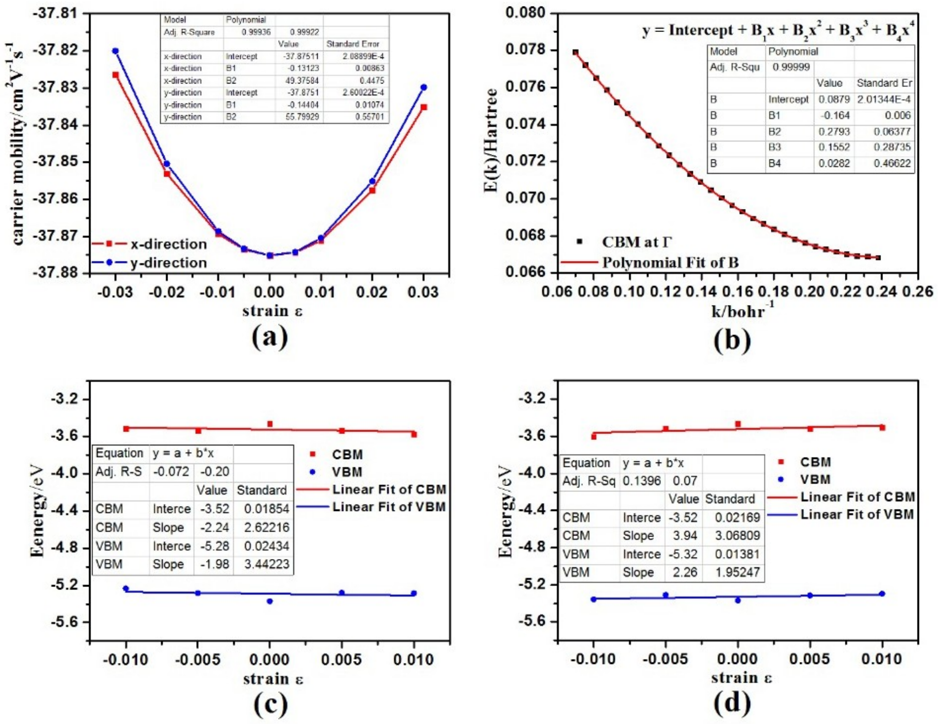

Fig S4 the Sc_2CF_2-I: the relationship between (a) the total energy and uniaxial strain along x direction and y direction, (b) The energy *E(k)* and the wave vector *k* near the CBM along x direction. Band edge position shift with respect to the uniaxial strain along (c) x direction, (d) y direction. The red and blue solid lines are the fittings to the data.

Table S1 Vacuum level, CBM and VBM levels (in V) for $Sc_2C(OH)_2$-I and Sc_2CF_2-I MXenes. E_{CBM} and E_{VBM} are band edge positions, which are listed relative to the Fermi level, vacuum level and normal hydrogen electrode (E_{NHE} = -4.5 V), respectively.

	Vacuum level	E_{CBM}			E_{VBM}		
		Vs fermi	*Vs* vacuum	*Vs* NHE	*Vs* fermi	*Vs* vacuum	*Vs* NHE
$Sc_2C(OH)_2$-I	1.472	0.701	-0.763	-3.737	-0.105	-1.568	-2.932
Sc_2CF_2-I	2.258	1.805	-3.462	-1.038	-0.103	-5.37	0.87

Table S2 Effective mass $|m^*|$, DP constant E_l, xy-plane elastic modulus C, and carrier mobility μ for electrons and holes along the x and y directions in the $Sc_2C(OH)_2$-I and Sc_2CF_2-I MXene.

		$m^*(m_e)$	$C(N/m)$	$E_l(eV)$	$\mu(cm^2V^{-1}S^{-1})$
$Sc_2C(OH)_2$-I	Electrons(x)	0.38	170.48	3.28	1558
	Hole(x)	1.65	170.48	-4.28	46
	Electrons(y)	0.38	192.24	3.92	1230
	Hole(y)	1.65	192.24	4.92	41
Sc_2CF_2-I	Electrons(x)	0.62	169.43	-2.24	1288
	Hole(x)	2.1	169.43	-1.98	139
	Electrons(y)	0.58	191.47	3.94	520
	Hole(y)	2.5	191.47	2.26	85

Computational Details of the Carrier Mobility:

As an example, we give the calculation process of the electron carrier mobility μ along x direction of the Sc_2CF_2-I. To obtain the elastic modulus C and deformation potential constant E_1, we calculated the total energy and band edge positions as a function of the uniaxial strain å as shown in Fig. S4 a, c and d. All the calculated results are listed in Table S2.

As for the effective mass, we plot a curve about the relationship between the energy *E(k)* and the wave vector *k* with thirty high-density k points near the CBM (Fig. S4 b), then adopted a fourth order polynomial function to fit this curve:

$$y = Intercept + B_1 x + B_2 x^2 + B_3 x^3 + B_4 x^4$$

The second derivative value of the fitting function:

$$\left.\frac{\partial^2 y}{\partial x^2}\right|_{x=M} = 2B_2 + 6B_3 x + 12B_4 x^2\Big|_{x=M}$$

According to the definition of carrier effective mass, the electron effective mass along x direction can be calculated.

$$m^* = \hbar^2 / (\partial^2 E(k) / \partial k^2)$$
